# Retrospective analysis of anamnesis, clinical signs, therapy, and outcome in 342 dogs with acute *Babesia canis* infections

**DOI:** 10.1016/j.crpvbd.2026.100407

**Published:** 2026-06-29

**Authors:** Clara M. Eisenecker, Andreas Moritz, Imke M. von Hohnhorst, Christina Strube, Elisabeth Müller, Ingo Schäfer

**Affiliations:** a Small Animal Clinic – Internal Medicine, Department of Veterinary Medicine, Justus-Liebig-University, Giessen, Germany; b Clinical Pathology and Clinical Laboratory Diagnostics, Department of Veterinary Medicine, Justus-Liebig-University, Giessen, Germany; c Institute for Parasitology, Centre for Infection Medicine, University of Veterinary Medicine Hannover, Hanover, Germany; d Laboklin GmbH & Co. KG, Bad Kissingen, Germany

**Keywords:** Canine babesiosis, Imidocarb dipropionate, Piroplasms, Thrombocytopenia, Ticks, Tick-borne diseases

## Abstract

*Babesia canis* is a protozoan vector-borne pathogen causing infections in dogs. Acute *B. canis* infections are emerging in central and eastern Europe. Clinicopathological abnormalities include fever, thrombocytopenia, and pigmenturia. This retrospective study aimed to analyse anamnesis, stays abroad, clinicopathological abnormalities, therapy, and outcome in dogs with acute *B. canis* infections in Germany. Three hundred and forty-two dogs with positive piroplasmid-PCR results (*B. canis* identified after sequencing) and negative *Anaplasma phagocytophilum-*PCR results between January 2018 and December 2024 were included if data on hematocrit, platelets, and leukocytes were available. Information about anamnesis, clinical findings, therapy, and outcome was collected through questionnaires. Acute *B*. *canis* infections occurred mainly in autumn (218/342; 63.7%), followed by spring (67/342; 19.6%), winter (34/342; 9.9%), and summer (23/342; 6.7%). Most dogs tested positive in northern federal states (259/342; 75.7%), with Berlin/Brandenburg, Saxony, Saxony-Anhalt, the Saarland, the Rhine-Main area and the Ruhr area classified as high-risk areas. Lethargy (147/220; 66.8%) and fever (127/228; 55.7%) were most frequently reported. Pigmenturia was detected in 53/204 dogs (26.0%). All 164 dogs with available information on treatment received imidocarb dipropionate (median dosage 3.3 mg/kg; 1.7–6.8 mg/kg body weight). An uncomplicated course of disease was reported in most dogs (169/193, 87.6%). The mortality rate was 6.7%. Acute *B. canis* infections are most often uncomplicated if prompt treatment is initiated and represent a differential diagnosis in case of thrombocytopenia throughout the year, even when presented without fever and pigmenturia, especially in high-risk areas.

## Highlights

• *Babesia canis* infection should not be ruled out in dogs without fever and hemoglobinuria.
• Rarely, no clinical signs are seen in dogs with acute *Babesia canis* infections.
• Identification of subclinical infections is important to prevent the spread of the pathogen.
• Prognosis is good if diagnosed early and treated adequately.

## Graphical abstract

**Figure:**
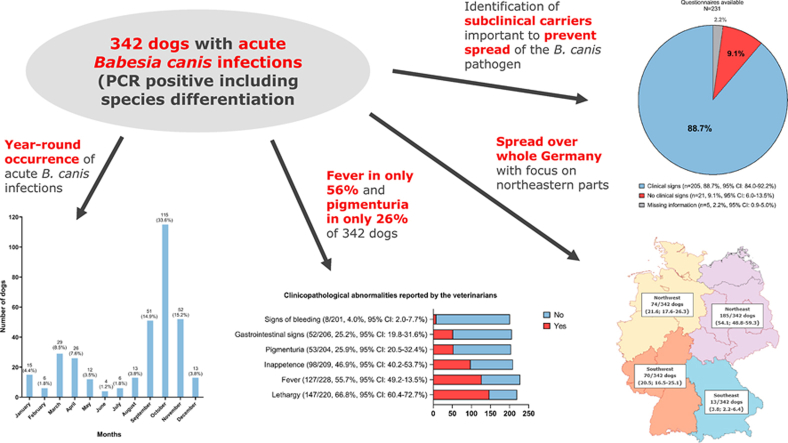

## 1 Introduction

*Babesia canis* is a protozoan vector-borne pathogen transmitted by *Dermacentor reticulatus* ticks. First autochthonous infections in Germany were described in the 1990’s, and infections were primarily diagnosed in dogs with stays abroad (Glaser and Gothe, 1998a, 1998b, b; Zahler et al., 2000a, 2000b). In recent years, the number of autochthonous *B*. *canis* infections reported in Germany has increased, which is closely linked to the expansion of *D. reticulatus* (Seibert et al., 2022; Schäfer et al., 2023; Weingart et al., 2023; Eisenecker et al., 2026). Climate change, increased import, travel of pets, and re-/deforestation are proposed factors contributing to the tick’s regional expansion (Gray et al., 2009; Mierzejewska et al., 2015, 2016; Diakou, 2024). *Dermacentor reticulatus* ticks were recently detected in all German federal states (Drehmann et al., 2020; Springer et al., 2022). Year-round *D. reticulatus* activity and year-round *B. canis* infections were documented over all parts of Germany (Springer et al., 2022; Probst et al., 2023a, 2023b; Schäfer et al., 2023). Three different *B. canis* genotypes with different outcomes on the severity of clinicopathological abnormalities were reported in Europe (Carcy et al., 2015). A broader genetic heterogeneity, based on the polymorphism of the *Bc28.1* gene, was demonstrated in dogs in the German federal states Berlin and Brandenburg (Helm et al., 2022), but the impact of these genotypes on individual severity of clinicopathological abnormalities in dogs with acute *B. canis* infections remains still poorly understood.

Clinical signs in acute *B. canis* infections are most often nonspecific. Lethargy (79–100%), fever (50–69%), and pale mucous membranes (2–50%), as well as hemolytic anemia and pigmenturia were reported in *B. canis*-infected dogs in Germany (Seibert et al., 2022; Weingart et al., 2023; Eisenecker et al., 2026). Thrombocytopenia (85–100%), anemia (79–85%), leukopenia (50–64%), hyperbilirubinemia (74–90%), increased liver enzyme activities (22–80%), hypoproteinemia (11–51%), and azotemia (increased creatinine: 17–55%) were most frequently documented in German dogs (Seibert et al., 2022; Weingart et al., 2023; Eisenecker et al., 2026). Further complications such as systemic inflammatory response syndrome (SIRS), disseminated intravascular coagulopathy (DIC), acute renal failure, multi-organ dysfunction syndrome (MODS), and neurological clinical signs were additionally described in dogs with acute *B. canis* infections (Matijatko et al., 2010; Adaszek et al., 2012; Dubova et al., 2023)

Molecular detection of *B. canis* *via* polymerase chain reaction (PCR) on EDTA-blood has high sensitivity and specificity and enables species differentiation (Jefferies et al., 2003; Solano-Gallego et al., 2016). Microscopic blood smear analysis has lower specificity and sensitivity compared to molecular detection (Böhm et al., 2006; Solano-Gallego et al., 2016; Schetters, 2019).

Subcutaneous application of 6.6 mg/kg body weight (BW) applied twice in a timeframe of 14 days was recently recommended by the Food and Drug Administration in the USA (FDA, 2026). Recent data indicate that one injection of 6.6 mg/kg BW imidocarb dipropionate with monitoring of the treatment success can achieve a negative PCR result in most dogs (Eisenecker et al., 2026). Depending on regionality and individual *B. canis* strains, the reported mortality rate varies from 1.5% in southwestern Europe to up to 20% in central and northeastern Europe (Carcy et al., 2015). An analysis of data on imidocarb treatment failure in a case report revealed a link to lower-range dosages, comorbidities, and infection with *Babesia* spp. other than *B. canis* (Schäfer et al., 2026).

Because of the increasing cases of acute *B. canis* infections in dogs and the rising importance of autochthonous infections in central Europe, our aim was to re-evaluate the regional and seasonal distribution of dogs with acute *B. canis* infections in Germany, to describe the clinical signs at first presentation, and to evaluate treatment protocols, treatment success, and outcomes of the disease.

## 2 Materials and methods

The 342 dogs included in this study were previously analyzed in another study focussing on levels of *B. canis* parasitemia, acute phase responses, and serological status (von Hohnhorst et al., 2025). In addition to these published data, the present study aimed to provide an in-depth analysis of the cohort’s anamnesis, clinicopathological findings, treatment, and outcome. The same inclusion and exclusion criteria with positive piroplamid-PCR testing (forward primer: 5′-AAT ACC CAA TCC TGA CAC AGG G-3′; reverse primer: 5′-TTA AAT ACG AAT GCC CCC AAC-3′, based on Olmeda et al. (1997)), revealing *B. canis* after sequencing, as well as negative *Anaplasma phagocytophilum* PCR-testing (real-time PCR, target: hsp60 gene) on EDTA blood, were applied to this study. For each of the 342 dogs with acute *B. canis* infections between January 2018 and December 2024, a questionnaire was sent to the primary care veterinarian, asking for the signalment, travel anamnesis, known tick prophylaxis, tick infestation, clinical signs, therapy, outcome, and underlying diseases. This information was ascertained through open-ended and closed-ended (binary) questions. Additional information was also queried by phone in some cases.

Seasons were categorized as follows: spring (March to May); summer (June to August); autumn (September to November); and winter (December to February). Geographical distribution was classified based on the federal states in northeastern (Berlin-Brandenburg, Saxony-Anhalt, Saxony, Mecklenburg-Western Pomerania, Thuringia), northwestern (Lower Saxony, North Rhine-Westphalia, Schleswig-Holstein), southwestern (Hesse, Baden-Württemberg, Rhineland-Palatinate, Saarland), and southeastern (Bavaria) Germany.

For statistical descriptive analysis, the software SPSS (v.30.0, IBM) was used. The 95% confidence intervals (CIs) were calculated for the most important results using the Wilson procedure, with continuity correction. The figures were created using GraphPad Prism v.10.5.0(774) for Windows.

## 3 Results

### 3.1 Survey response rate

Questionnaires were completed for 231 of 342 dogs (67.5%). By extending the query by phone, certain information could be completed and/or expanded (e.g. clinical signs). In 105 of 342 cases (30.7%), no information could be gathered by questionnaire or phone. In some cases, information was only partially available.

### 3.2 Patients

**Fig. 1:**
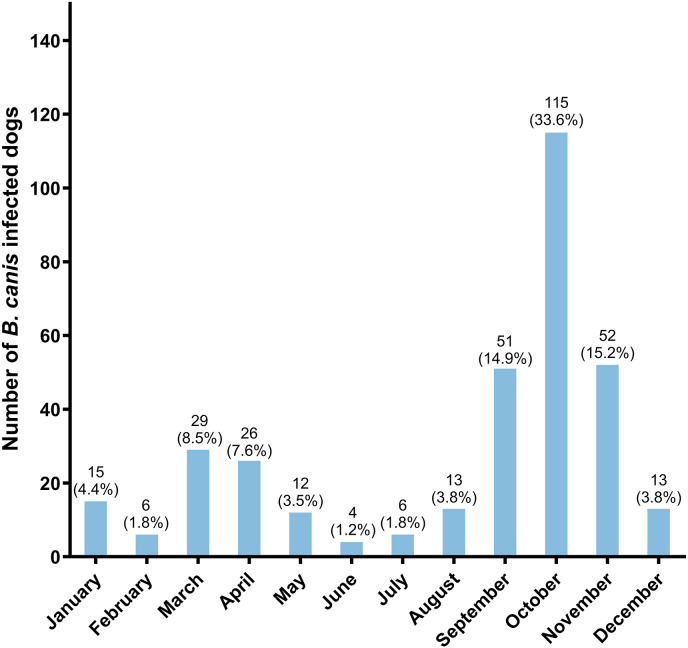
*Babesia canis* PCR diagnosis by month in 342 dogs from January 2018 to December 2024 in Germany. The number of infected dogs per month is shown above individual bars, with percentages in parentheses.

This retrospective study included 342 dogs as previously described in another manuscript by our study group (von Hohnhorst et al., 2025). Most dogs were diagnosed with acute *B. canis* infection in autumn (218/342; 63.7%, 95% CI: 58.5–68.7%), followed by spring (67/342; 19.6%, 95% CI: 15.7–24.1%), winter (34/342; 9.9%, 95% CI: 7.2–13.6%), and summer (23/342; 6.7%, 95% CI: 4.5–9.9%). The highest percentage of positive PCR results for *B. canis* was recorded in October (115/342; 33.6%, 95% CI: 28.8–38.8%), and the lowest in June (4/342; 1.2%, 95% CI: 0.5–3.0%) (Fig. 1).

**Fig. 2:**
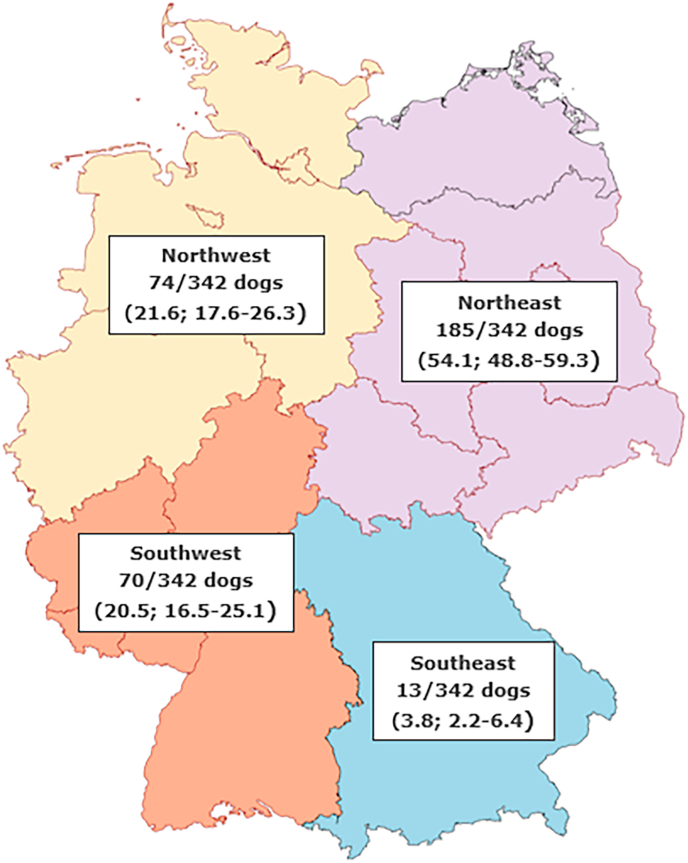
Regional distribution of 342 dogs diagnosed with acute *Babesia canis* infections by PCR from January 2018 to December 2024 in Germany (%; lower 95% CI - upper 95% CI).

Most dogs were diagnosed in the northeast of Germany (185/342; 54.1%, 95% CI: 48.8–59.3%), followed by the northwest (74/342; 21.6%, 95% CI: 17.6–26.3%), the southwest (70/342; 20.5%, 95% CI: 16.5–25.1%), and the southeast (13/342; 3.8%, 95% CI: 2.2–6.4%) (Fig. 2). Most of the samples were sent from Berlin and Brandenburg (128/342; 37.4%, 95% CI: 32.5–42.7%), Lower Saxony (44/342; 12.9%, 95% CI: 9.7–16.8%), Hesse (27/342; 7.9%, 95% CI: 5.5–11.2%), and Saxony Anhalt (26/342; 7.6%, 95% CI: 5.2–10.9%) (Table 1).

**Table 1:** Federal states for 342 dogs diagnosed with an acute *Babesia canis* infection by piroplasm PCR with species differentiation between January 2018 and December 2024 in Germany.

Region	Federal state	*n*/*N*	Percent of total (95% CI)
Northeast	Berlin and Brandenburg	128/342	37.4 (32.5–42.7)
	Saxony	13/342	3.8 (2.2–6.3)
	Saxony-Anhalt	26/342	7.6 (5.2–10.9)
	Thuringia	4/342	1.2 (0.5–3.0)
	Mecklenburg-Western Pomerania	14/342	4.1 (2.5–6.8)
Northwest	Lower Saxony	44/342	12.9 (9.7–16.8)
	North Rhine-Westphalia	23/342	6.7 (4.5–9.9)
	Schleswig-Holstein	7/342	2.0 (1.0–4.2)
Southwest	Hesse	27/342	7.9 (5.5–11.2)
	Baden-Wuerttemberg	22/342	6.4 (4.3–9.5)
	Rhineland Palatinate	10/342	2.9 (1.6–5.3)
	The Saarland	10/342	2.9 (1.6–5.3)
Southeast	Bavaria	14/342	4.1 (2.5–6.8)
Total		342/342	100

*Abbreviations*: *n*, number tested positive; *N*, total number; CI, confidence interval.

### 3.3 Anamnesis and clinical signs

Information on the history of stays abroad was available in 191 of 342 dogs (55.8%, 95% CI: 50.6–61.0%). One hundred and thirteen of 191 dogs (59.2%, 95% CI: 52.1–65.9%) had no history of stays abroad, while 55 (28.8%, 95% CI: 22.8–35.6%) were imported. Twenty-three of 191 dogs (12.0%, 95% CI: 8.2–17.4%) had previously travelled abroad, as previously described in von Hohnhorst et al. (2025).

Information about tick infestation was available for 133 of 342 dogs (38.9%, 95% CI: 33.8–44.2%). In 104 of 133 dogs (78.2%, 95% CI: 70.4–84.4%), ticks were detected during the clinical examination.

**Fig. 3:**
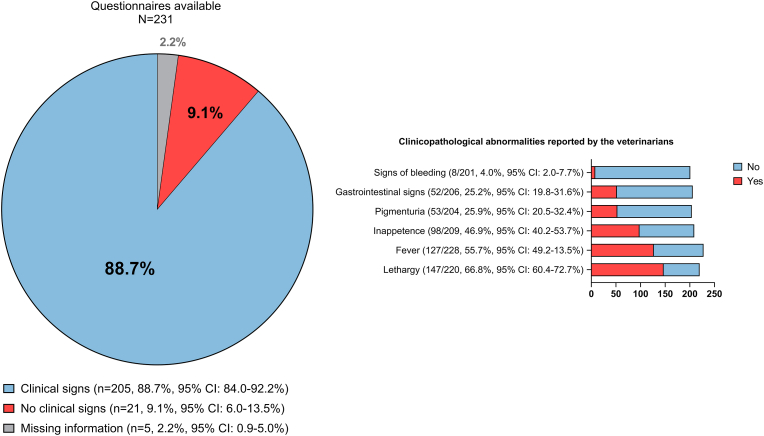
Clinicopathological abnormalities in 231 dogs diagnosed with acute *Babesia canis* infections by PCR from January 2018 to December 2024 in Germany (%; lower 95% CI - upper 95% CI).

For 231 of 342 dogs (67.5%, 95% CI: 62.4–72.3%), the treating veterinarian completed the questionnaire. Two-hundred-and-five of these 231 dogs (88.7%, 95% CI: 84.0–92.2%) showed clinical signs with lethargy (147/220; 66.8%, 95% CI: 60.4–72.7%) fever (127/228; 55.7%, 95% CI: 49.2–62.0%), inappetence (98/209; 46.9%, 95% CI: 40.2–53.7%), and pigmenturia (53/204; 26.0%, 95% CI: 20.5–32.4%) as the most frequent findings (Fig. 3). Of the 53 dogs with pigmenturia, 19 (35.8%) were from Berlin-Brandenburg, followed by Lower Saxony (9/53; 17.0%) and Saxony-Anhalt (7/53; 13.2%). Of the dogs from the Berlin-Brandenburg subpopulation, 15 of 56 (26.7%) showed pigmenturia. Twenty-one of 231 dogs (9.1%, 95% CI: 6.0–13.5%) did not show any clinical signs. Of these, information about stays abroad was available for 20 dogs, of which 18 (90%) had been imported, one (5%) had travelled, and one (5%) had no stays abroad.

### 3.4 Treatment and outcome

Data regarding treatment were available for 164 of 342 dogs (48.0%, 95% CI: 42.7–53.2%). Of these, 161 dogs (98.2%, 95% CI: 94.8–99.4%) received either one (24/164; 14.6%, 95% CI: 10.0–20.9%) or two (137/164; 83.5%, 95% CI: 77.1–88.4%) imidocarb dipropionate injections. Three dogs (1.8%, 95% CI: 0.6–5.2%) were administered three injections, and one dog (0.6%, 95% CI: 0.1–3.4%) received four injections. The imidocarb dipropionate injections ranged from 1.7 mg/kg BW to 6.8 mg/kg BW (median: 3.3 mg/kg BW).

Information regarding outcome was available for 193 of 342 dogs (56.4%, 95% CI: 51.1–61.6%). Uncomplicated courses were reported in 169 of 193 dogs (87.6%, 95% CI: 82.2–91.5%), complicated courses in 24 of 193 dogs (12.4%, 95% CI: 8.5–17.8%). Thirteen dogs died (mortality rate: 6.7%, 95% CI: 4.0–11.2%). In dogs without stays abroad, information on the outcome was available in 101 of 113 dogs (89.4%, 95% CI: 82.4–93.8%). In this group, complicated course with survival was described for 7 dogs (6.9%, 95% CI: 3.4–13.6%), and complicated courses with subsequent death or euthanasia were reported for another 7 dogs, resulting in a mortality rate of 6.9% (95% CI: 3.4–13.6%). Of the 20 dogs that had travelled, 3 died (mortality rate: 15.0%). Of the 40 imported dogs with available information on the outcome, all had uncomplicated courses and a 100% survival rate.

## 4 Discussion

This retrospective study on 342 dogs with acute *B. canis* infections includes the largest cohort in Europe to date. Clinicopathological abnormalities in these dogs were in general in accordance with literature (Seibert et al., 2022; Weingart et al., 2023). However, fever was only reported in 55.7% and pigmenturia in 26.0% of dogs in our study. Acute *B. canis* infections should therefore be considered as a potential differential diagnosis even if fever and/or pigmenturia are absent, especially if thrombocytopenia is present. Thrombocytopenia was detected in all 342 dogs and was most often marked with platelet counts < 40 G/l (von Hohnhorst et al., 2025). Granulocytic anaplasmosis as another endemic disease in Germany caused by *A. phagocytophilum*, with thrombocytopenia as the most frequent hematological abnormality (Chirek et al., 2018; Schäfer et al., 2022) was ruled out by negative PCR-testing, as *A. phagocytophilum* infections are a differential diagnosis with similar clinical presentation and clinicopathological abnormalities. The identified federal states with high proportions of positive samples are in accordance with another study in Germany and include Berlin and Brandenburg, Saxony, Saxony-Anhalt, the Ruhr area, the Saarland, and the Rhine-Main area (Seibert et al., 2022; Schäfer et al., 2023; Weingart et al., 2023). Bias can be due to a low number of samples in individual federal states or due to the fact that veterinarians in high-risk areas have become familiar with the disease and therefore did not submit samples for confirmation to a diagnostic laboratory (IS, personal observation). The Saarland, for example, did not reveal a high rate of positive dogs; however, canine *B. canis* infections have been recognized for decades in this area, and 2.5% of ticks tested were positive for *B. canis* (Beelitz et al., 2012). Veterinarians in this area are familiar with the disease and may, therefore, diagnose *B. canis* infections by detection of merozoites in capillary blood smears without sending a sample for PCR-testing to a commercial laboratory, especially in cases of tick infestation, thrombocytopenia, and/or anemia (IS, personal observation).

As PCR-positive dogs may transmit *B. canis* to ticks during the blood meal and might therefore contribute to the spread of the pathogen, dogs with thrombocytopenia should be checked for *B. canis* infections even if no clinical signs are detected. It is also recommended to screen blood donors for *B. canis* infections by PCR, as the pathogen can be transmitted by blood transfusions (Solano-Gallego et al., 2016). The occurrence and severity of clinical signs in acute *B. canis* infections might be linked to the *Babesia* spp. antibody levels and pre-existing immunity. Dogs with higher antibody levels showed less pronounced levels of *B. canis* parasitemia, acute phase reactions, and hematological abnormalities (von Hohnhorst et al., 2025). This was also seen in the Pirodog® vaccination, in which antibody formation against *B. canis* was demonstrated to result in less severe clinical and clinicopathological outcomes of infections (Schetters et al., 1994; Schetters, 2005; Nathaly Wieser et al., 2019). Positive *Babesia* spp. antibody levels were demonstrated mainly in dogs with stays abroad, whereas dogs in Germany without any history of stays abroad were predominantly serologically naïve (von Hohnhorst et al., 2025). This finding could be corroborated by the fact that imported dogs showed only uncomplicated courses of the disease with a 100% survival rate in our study.

Pigmenturia and fever were previously described as typical clinical signs for acute *B. canis* infections (Mathe et al., 2006). Pigmenturia was detected in only 26% of patients in our cohort, suggesting less intravascular hemolysis. In a previous study conducted in Berlin-Brandenburg, pigmenturia was observed in 49% of dogs presented with an acute *B. canis* infection (Weingart et al., 2023), while in our study, pigmenturia was reported in only 27% of dogs from this area. This may be linked to the fact that 93.9% of the dogs included in the study by Weingart et al. (2023) were presented to a university clinic and were most likely referrals. These dogs might have already shown a prolonged course of disease compared to the dogs in our study, which were predominantly presented to primary-care veterinarians. Additionally, veterinarians might consider *B. canis* infections as a differential diagnosis for clinical signs earlier due to the rise in cases in Germany, and owners might visit the veterinarian earlier in case of the occurrence of clinical signs (Schäfer et al., 2023). Thrombocytopenia is, in general, the first hematological abnormality followed by leukopenia and hemolytic anemia leading to hemoglobinuria in experimental acute *B. canis* infections (Schetters et al., 2009). All of these may explain the lower percentage of dogs presented with pigmenturia and anemia in the present study, as well as the predominance of dogs with thrombocytopenia.

We confirmed the trend for year-round occurrence of acute *B. canis* infections and the regional spread in Germany, which was also recorded in other recent studies (Seibert et al., 2022; Schäfer et al., 2023; Weingart et al., 2023). Although distinct peaks in spring and especially in autumn remained apparent, acute *B. canis* infections were diagnosed year-round. Milder temperatures due to climate change are favourable for the tick’s activity in colder months, while deforestation and reforestation with subsequent expansion of the wildlife population represent an increase in hosts for the tick vector (Gray et al., 2009; Mierzejewska et al., 2015, 2016; Probst et al., 2023a, 2023b). The increasing travel of humans and pets significantly contributes to the spread of diseases and ticks (Diakou, 2024; Springer et al., 2024). This underlines the necessity of year-round, licensed protective measures against ticks. With 90% of asymptomatic dogs being imported, the possibility of imported dogs functioning as reservoir hosts for *B. canis* should be considered, as already described in the 1990’s (Glaser and Gothe, 1998a, 1998b).

Most dogs received two imidocarb dipropionate injections as recommended by the FDA (FDA, 2026). With the median dosage being 3 mg/kg, many dogs likely received the dosage indicated in the European Carbesia® package insert (2.125 mg/kg) (MSD Animal Health GmbH, 2021). The mentioned dosages have been established for France as an area endemic for less pathogenic *B. canis* strains, whereas the current FDA-approved recommendation is 6.6 mg/kg, with two injections subcutaneously or intramuscularly at the time of diagnosis and 14 days later (FDA, 2026). Lower-ranged imidocarb dipropionate dosages in combination with glucocorticoids were suggested to be one major underlying cause for potential treatment failure in dogs with acute *B. canis* infections, complicating the course of disease (Schäfer et al., 2026). Complicated courses were described for 12.4% of dogs in the present study, including a mortality rate of 6.7%. Compared to recent studies in Germany with mortality rates up to 15% (Weingart et al., 2023), the higher mortality rate may be linked to the already discussed hypothesis that most cases might have been referrals with a more complicated course of disease compared to the present study, including predominantly primary-care veterinarian service. Another influencing factor might be pre-existing immunity, but antibody levels were not available in the study by Weingart et al. (2023). The mortality rates may additionally be influenced by different *B. canis* genotypes (Carcy et al., 2015), out of which a broad heterogenicity was detected in one study in Germany (Helm et al., 2022). As no genotyping was included in the present study, the impact of genotypes on the severity of clinical signs and the outcome remains unknown.

Limitations of the present study are linked to the retrospective design and the fact that information provided by the treating veterinarian was not complete for all 342 dogs. Missing and incomplete data, especially for anamnesis of stays abroad, clinical signs, treatment information, and outcome, reduce the representativeness of the study to a certain extent. Furthermore, high regional percentages of dogs diagnosed with a *B. canis* infection do not necessarily indicate high-risk areas, as the samples were sent by veterinarians for diagnostic purposes, which implies clinical suspicion of babesiosis in many cases. Therefore, the present data most likely overestimate the true prevalence of babesiosis in Germany. On the other hand, high-risk areas such as the Saarland with veterinarians familiar to the disease may be underrepresented due to diagnostic work being completely done in clinical practice without submissions to a diagnostic laboratory. Finally, only *A. phagocytophilum* was ruled out as a possible co-infection, not taking into consideration other pathogens.

## 5 Conclusions

Acute *B. canis* infections occurred year-round in Germany, with a marked peak in autumn. Consistent with previous reports, most affected dogs exhibited nonspecific clinical signs, most commonly lethargy and inappetence. A small proportion of dogs remained clinically asymptomatic despite PCR-confirmed acute *B. canis* infections. Treatment is indicated in these cases to prevent further pathogen transmission. Acute *B. canis* infection should not be excluded in dogs lacking fever and/or hemoglobinuria, particularly in the presence of tick infestation and thrombocytopenia in endemic areas. Imidocarb dipropionate treatment was generally effective despite substantial variation in administered dosages. The overall prognosis was favourable, with a mortality rate of 7%. None of the dogs imported from other countries to Germany died, probably due to a pre-existing immunity, with antibodies protective against severe clinicopathological abnormalities.

## Ethical approval

Not applicable.

## CRediT authorship contribution statement

**Clara M. Eisenecker:** Investigation, Visualization, Writing - original draft. **Andreas Moritz:** Supervision, Writing - review & editing.**Imke M. von Hohnhorst:** Writing - review & editing. **Christina Strube:** Writing - review & editing. **Elisabeth Müller:** Writing - review & editing. **Ingo Schäfer:** Conceptualization, Supervision, Writing - review & editing.

## Funding

This research did not receive any specific grant from funding agencies in the public, commercial, or not-for-profit sectors.

## Declaration of competing interests

The authors declare the following financial interests/personal relationships which may be considered as potential competing interests: Ingo Schäfer is an employee, and Elisabeth Müller is the CEO of Laboklin GmbH & Co. KG; Andreas Moritz, Christina Strube, and Ingo Schäfer have repeatedly lectured for and/or acted as consultants for diagnostic and (veterinary) pharmaceutical companies; Andreas Moritz and Christina Strube have previous and ongoing research collaborations with various diagnostic and (veterinary) pharmaceutical companies. The other authors declare that they have no known competing financial interests or personal relationships that could have appeared to influence the work reported in this paper.

## Data Availability

All data generated or analyzed during this study are included in this published article.
